# Methodological approach for the assessment of ultrasound reproducibility of cardiac structure and function: a proposal of the study group of Echocardiography of the Italian Society of Cardiology (Ultra Cardia SIC) Part I

**DOI:** 10.1186/1476-7120-9-26

**Published:** 2011-09-26

**Authors:** Maurizio Galderisi, Stefano Nistri, Sergio Mondillo, Maria-Angela Losi, Pasquale Innelli, Donato Mele, Denisa Muraru, Antonello D'Andrea, Piercarlo Ballo, Aurelio Sgalambro, Roberta Esposito, Giuliano Marti, Alessandro Santoro, Eustachio Agricola, Luigi P Badano, Roberto Marchioli, Pasquale Perrone Filardi, Giuseppe Mercuro, Paolo Nicola Marino

**Affiliations:** 1 Department of Clinical and Experimental Medicine, Federico II University Hospital, Via S. Pansini 5, 80131, Naples, Italy; 2 CMSR Veneto Medica, Via Vicenza 204, 36077, Altavilla Vicentina (Vicenza), Italy; 3 Department of Cardiovascular Diseases, "Le Scotte" University Hospital, 53100 Siena, Italy; 4 Department of Clinical Medicine, Cardiovascular and Immunological Sciences, Federico II University Hospital, Via S. Pansini 5, 80131 Naples, Italy; 5 Division of Cardiology, Hospital of Villa d'Agri. Via Provinciale, 85050 Villa d'Agri (Potenza), Italy; 6 Department of Cardiology, University of Ferrara,, Corso Giovecca 203, 44100 Ferrara, Italy; 7 Department of Cardiology, Vascular and Thoracic Sciences, University of Padua, Via Giustiniani 2, 35100 Padua, Italy; 8 Department of Cardiology, Second University of Naples, "dei Colli - Monaldi" Hospital, Via L. Bianchi, 80131, Naples, Italy; 9 Department of Cardiology, S. Maria Annunziata Hospital, Via dell'Antella 58, 50100 Florence, Italy; 10 Department of Cardiology, Careggi University Hospital, 50141 Florence, Italy; 11 Clinical Cardiology, University of Eastern Piedmont, "Maggiore della Carità" University Hospital, Corso Mazzini 18, 28100 Novara, Italy; 12 Division of Cardiology, Non invasive diagnostics, Cardio-Thorax-Vascular Department San Raffaele Hospital, Via Olgettina 60, 20132 Milan, Italy; 13 Research Centre, Italian Society of Cardiology, Via Po' 24, 00198, Roma, Italy

**Keywords:** Doppler echocardiography, Clinical trials, Quality control, Reproducibility, Echo core laboratory

## Abstract

When applying echo-Doppler imaging for either clinical or research purposes it is very important to select the most adequate modality/technology and choose the most reliable and reproducible measurements. Quality control is a mainstay to reduce variability among institutions and operators and must be obtained by using appropriate procedures for data acquisition, storage and interpretation of echo-Doppler data. This goal can be achieved by employing an echo core laboratory (ECL), with the responsibility for standardizing image acquisition processes (performed at the peripheral echo-labs) and analysis (by monitoring and optimizing the internal intra- and inter-reader variability of measurements). Accordingly, the Working Group of Echocardiography of the Italian Society of Cardiology decided to design standardized procedures for imaging acquisition in peripheral laboratories and reading procedures and to propose a methodological approach to assess the reproducibility of echo-Doppler parameters of cardiac structure and function by using both standard and advanced technologies. A number of cardiologists experienced in cardiac ultrasound was involved to set up an ECL available for future studies involving complex imaging or including echo-Doppler measures as primary or secondary efficacy or safety end-points. The present manuscript describes the methodology of the procedures (imaging acquisition and measurement reading) and provides the documentation of the work done so far to test the reproducibility of the different echo-Doppler modalities (standard and advanced). These procedures can be suggested for utilization also in non referall echocardiographic laboratories as an "inside" quality check, with the aim at optimizing clinical consistency of echo-Doppler data.

## Introduction

Echo-Doppler examination is the most commonly used non invasive cardiac imaging technique in the clinical practice for evaluating the effects of diseases and/or treatment on cardiac function. Accuracy, reliability and reproducibility of echo-Doppler measurements represent main goals to address appropriately diagnosis, decision making and reduce the frequency of unnecessary, repeated examinations. Echo-Doppler is also widely applied in clinical trials in order to identify potential mechanisms of clinical end-points or to assess surrogate end-points [1-3]. This use must be considered in the context of the overall trial, the possible regulatory requirements and the role that imaging might fill [3]. When applying echo-Doppler in clinical trials it is mandatory to select the most adequate echo-Doppler modalities and measurements to answer the specific question for which a given trial has been designed. This choice is largely dependent on the various sources of acquisition and measurement variability which can result into inaccuracy of collecting data. In this view, the improvement of reproducibility of echo Doppler measurements is pivotal to guarantee a high level results. This goal can be achieved by employing an echo core laboratory (ECL), with the objective of producing enough robust data to support or discard the hypothesis for which a given trial has been designed. ECL shall have the responsibility of ensuring the best data quality by standardizing image acquisition processes at peripheral echo-labs and minimizing measurement variability, i.e., by monitoring the inside inter-reader reproducibility [1-3]. The role of ECL will depend on the type of trial, the complexity of analyses required and how the data collected will be analyzed/interpreted, but also on the regulatory oversight involved. The American Society of Echocardiography (ASE) has defined three broad categories of echocardiography use in clinical trials according to the presence (category A) or absence (categories B and C) of FDA or other regulatory body oversight [3].

The Working Group of Echocardiography of the Italian Society of Cardiology decided to design standardized procedures for imaging acquisition in peripheral laboratories and reading procedures in ECLs in order to propose a methodological approach to assess the reproducibility of echo-Doppler parameters of cardiac structure and function derived from both standard and advanced modalities. A number of cardiologists experienced in cardiac ultrasound was involved to set up ECL procedures available for future studies involving complex imaging or echo-Doppler measures as primary or secondary efficacy or safety end-points (category B of ASE standards for ECL) [3].

The present manuscript describes this methodologic approach and provides the documentation of the work done so far to test the reproducibility of the different echo-Doppler (standard and advanced) modalities.

## Study project and methodology

**Figure 1:**
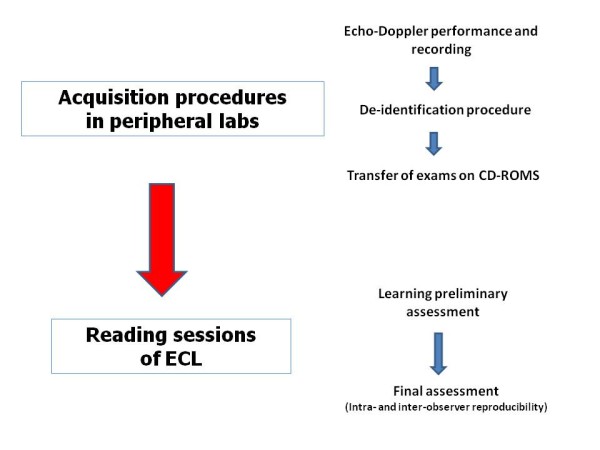
**Flow chart of the methodological approach for acquisition procedures of peripheral centers and reading sessions of echo core lab (ECL) suggested by the Study Group of Echocardiography of the Italian Society of Cardiology**.

The study project and methodology were designed and approved by the Nucleus of the Working Group of Echocardiography, and developed under the auspices of the Research Centre of the Italian Society of Cardiology. Figure 1 sumarizes the methodological approach used for imaging acquisition in the peripheral centers and reading procedures of ECL.

Fourteen board-certified cardiologists (from 10 different national laboratories) of the Working Group of Echocardiography of the Italian Society of Cardiology were preliminary invited to collect echocardiographic video clips and images in their peripheral labs during their daily activity according a predetermined protocol/agreement. All the patients undergoing echo Doppler examinations gave their written informed consent. Each peripheral lab was requested to store echo-Doppler exams from consecutive patients in sinus rhythm, without contraindications for analysis (e.g. massive calcification of the mitral annulus or mitral prosthesis for pulsed Tissue Doppler). Patients with suboptimal images quality were not included. Images and video clips were acquired, digitally stored on the machine hard disk and, after generating ID (de-identification procedure) through numerical codes, transferred on CD-ROMS as recommended [2,4]. The collection of a overall minimal number of 50 exams for each modality/technology was mandatory for the purposes of the project.

The same investigators met at the General Electric Learning Center of Naples, July 16-17, 2010. They were randomly divided in 7 couples of readers in order to test the intra- and inter-reading variability of the echo-Doppler data sets previously collected. All the analyses were performed using Echopac BT 09 work-stations (GE, Horten, Norway).

Echo-Doppler analyses tested for reproducibility are listed in Table 1. The proposed methodological approach of reading procedures (Table 2) included a preliminary assessment (3 steps) and a final assessment corresponding to the independent evaluation of the cases recorded for each echo-Doppler modality/technology by the two readers (twice for the reader # 1 in order to test intra-observer reproducibility) of each couple. *Inter-observer reproducibility *was defined as the reproducibility calculated by the two physicians' analyses of the same set of recordings. *Intra-observer reproducibility *was defined as the reproducibility calculated by one of the physicians re-doing his own measurements in a random order.

**Table 1:** List of echo Doppler analyses tested for reproducibility by the Echo Study Group of the Italian Society of Cardiology

Type of cardiac ultrasound analysis	
1	Quantitative analysis of the left ventricle

2	Quantitative analysis of left atrium, aortic root and ascending aorta

3	Quantitative analysis of the right ventricle

4	Doppler derived left ventricular diastolic function (including pulsed Tissue Doppler of the mitral annulus)

5	Speckle Tracking Echocardiography and AFI-derived LV longitudinal strain

6	Real time 3D echocardiography of the left ventricle

7	Real-time 3D echocardiography of the right ventricle

AFI = Automated function imaging, LV = Left ventricular

**Table 2:** Methodological approach of reading procedures for testing inter- and intra-observer variability of echo-Doppler parameters

Assessment Procedures	
**A. Preliminary assessment**	1. Joint discussion on how to measure parameters by using sample echo studies not included in subsequent analysis
	2. Preliminary reading session by the 2 readers of each couple on 4 cases for each echo-Doppler modality which were not included in the subsequent reproducibility analyses
	3. Reciprocal training by the readers and standardization of measurements

**B. Final assessment**	1. First and second reading in random order by one of the observers of each couple
	2. Blind independent reading of the second observer

### Technical Procedures

#### 1. Acquisition procedures (in the peripheral centers)

##### LV structure and function [2,5]

Recordings of the parasternal long-axis view (2-D or 2-D targeted M-mode images) were performed to obtain measurements of left ventricular (LV) diameters and wall thickness. Apical 4-chamber and 2-chamber views were recorded to measure volumes and ejection fraction (EF) by biplane Simpson's rule. To increase accuracy of 2-D volume measurements, the operators were requested to avoid LV cavity foreshortening by reducing the difference of the LV long-axes length in 4- and 2-chamber views < 10%. Images were acquired either during held expiration or quiet respiration to minimize translational motion of the heart. Depth setting was optimized to display the left ventricle on the screen as large as possible and the same field depth was kept for both 4- and 2-chamber apical views. Sector width was reduced to increase spatial and temporal resolution.

##### LA volumes [2,5]

**Figure 2:**
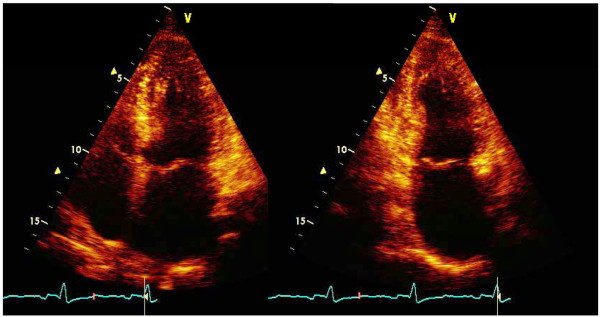
**2-D acquisition of apical 4-chamber and 2-chamber view for the measure of left atrial volume**. Quantification of LA volume was performed by apical approaches (4-chambers, left; 2-chambers, right) at end-systole (end of the T-wave of ECG trace), the frame before the opening of the mitral valve, maximizing LA length and area. Views were optimized reducing the sector angle width, and focusing the far filed in order to improve the wall definition without increasing the gain for better identification of LA walls.

From apical approaches, 4-chamber and 2-chamber apical views were purposely adjusted for computing left atrial (LA) volumes. In order to optimize consistency and reproducibility of measurements, peripheral labs were invited to record dedicated views to maximize LA length and area and not to use the same video-clips recorded for LV assessment (Figure 2). To avoid foreshortening, the difference of the two lengths (perpendicular from the mid-line of the plane of mitral annulus to the opposite superior part of the left atrium) in apical 4-chamber and 2-chamber views had to be < 5 mm.

##### Aortic root and ascending aorta [6]

**Figure 3:**
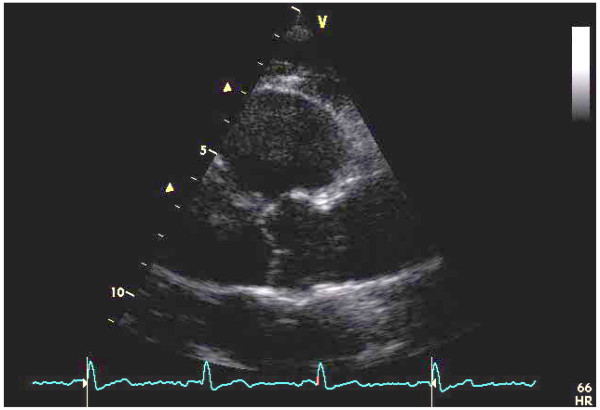
**Aortic root and proximal ascending aorta in parasternal long-axis view by 2-D echocardiography**. By parasternal approach, a long-axis view was modified in order to maximize the imaging of the aortic valve, the sinuses of Valsalva, the sino-tubular junction and the proximal ascending aorta at end-diastole. The probe was thus swept in order to make the whole aortic root as perpendicular as possible to the ultrasound beam. Gain settings, compensation and dynamic ranges were adjusted to optimize aortic wall definition.

Aortic root and proximal ascending aorta were recorded by 2-D echocardiography in parasternal long-axis view. Operators of peripheral labs were invited to record dedicated views aimed at obtaining the best visualization of the Valsalva sinuses, sino-tubular junction and proximal ascending aorta, until 2-3 cm above the sino-tubular junction (Figure 3).

##### Doppler derived LV diastolic function [2,7]

Mitral inflow velocities were recorded by pulse wave Doppler in the apical 4-chamber view. By the guide of colour flow imaging, a 1-mm to 3-mm sample volume was placed at the level of mitral leaflet tips where the signal amplitude is maximal.

Pulsed wave Tissue Velocity Imaging (TVI) was recorded in the apical 4-chamber view, with the sample volume placed at either the septal or the lateral insertion of the mitral annulus and adjusted as needed to cover the longitudinal annular excursion in systole as well as in diastole. When performing TVI the longitudinal excursion of LV wall was aligned with the Doppler beam. Attention was addressed to the Doppler spectral gain settings and the velocity scale was at about 20 cm/s above and below the baseline. Minimal angulation (< 20°) was maintained between the ultrasound beam and the plane of cardiac motion.

All Doppler recordings were obtained at end-expiration and at sweep speed of 50-100 mm/s, in order to improve temporal resolution and thus reproducibility of time interval measurements.

##### RV structure and function [2,5]

Quantitative assessment of right ventricular (RV) size was performed by 2-D echocardiography taking care to obtain a true non-foreshortened apical 4-chamber view, oriented to obtain the maximal RV area. M-mode tracings for the estimation of the tricuspid annular plane systolic excursion (TAPSE) were obtained during held respiration. Attention was paid to align M-mode along the direction of tricuspid annulus motion.

##### Speckle Tracking Echocardiography [8]

**Figure 4:**
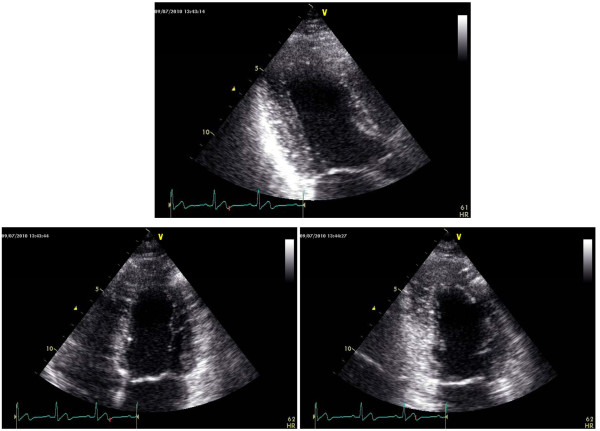
**2-D apical views at end-systole (upper panel: apical long-axis view, lower panels: apical 4-chamber view on the left, 2-chamber view on the right) for subsequent STE or AFI analysis**.

Speckle Tracking Echocardiography (STE) of the left ventricle was recorded on 3 consecutive cardiac cycles of 2-D images from apical views (long axis, 4- and 2-chamber) (Figure 4) and parasternal short-axis views (at base - just below the mitral level, at the mid (papillary muscle) level and at the apex - just proximal to the level with LV cavity obliteration at end-systole). Reliable recording of 2-D images for STE requires a high frame rate (40-70 frames/s), without dual focusing. To achieve this goal peripheral labs were requested to record LV cavity with the narrowest scan and at the lowest possible depth in order to display on the screen the left ventricle as large as possible. The same field depth was kept for all the views. Care was taken to record the video clips for subsequent STE analysis at an approximately equal heart rate.

## RT3DE

### Left ventricle

**Figure 5:**
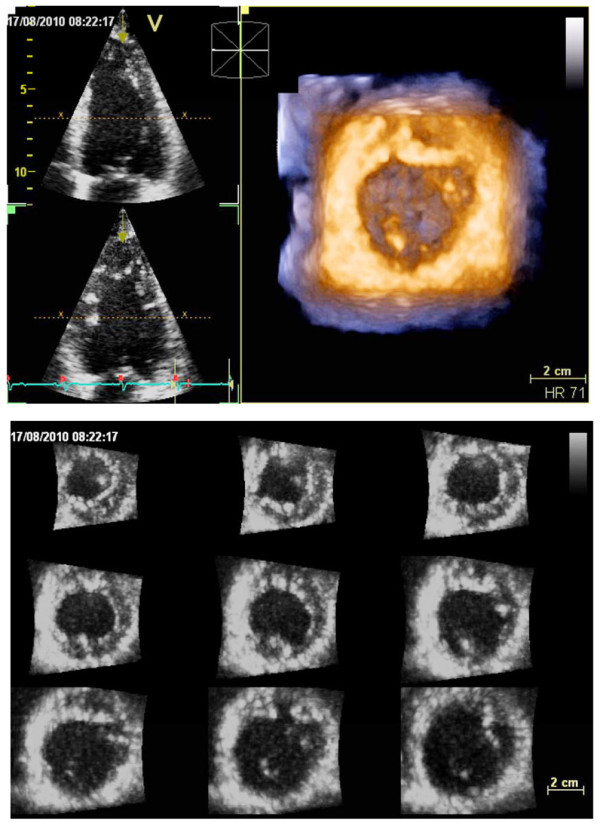
**Real time 3-D echocardiography for quantitation of the left ventricle**. Care was taken to encompass the entire LV cavity in the data set by checking LV views from 2-D multiplane display and 3-D LV transversal plane (upper panel). After the 3-D acquisition, 9-slice display mode was used to ensure optimal imaging of the entire LV endocardium at each short-axis level and lack of stitching artifacts (lower panel).

A full-volume LV data-set was acquired using harmonic imaging, with adjustment of image contrast, frequency, depth and sector size for adequate frame-rate and optimal LV border visualization. Mitral valve, but not the entire left atrium, was included in the data set throughout the cardiac cycle. Gain was set higher than for usual 2-D images. Four ECG-gated subvolumes were acquired from consecutive cardiac cycles during apnea to generate the full-volume data set. Peripheral labs were requested to perform "on-line" quality check to ensure that the entire LV cavity and wall thickness were included in the data set:

1. before the full volume acquisition by checking LV views from 2-D multiplane display and 3-D LV transversal plane (Figure 5);

2. after the full volume acquisition, by 9-slice display mode to ensure optimal imaging of the entire LV endocardium at each short-axis level and lack of stitching artifacts (Figure 5) [9].

### Right ventricle

**Figure 6:**
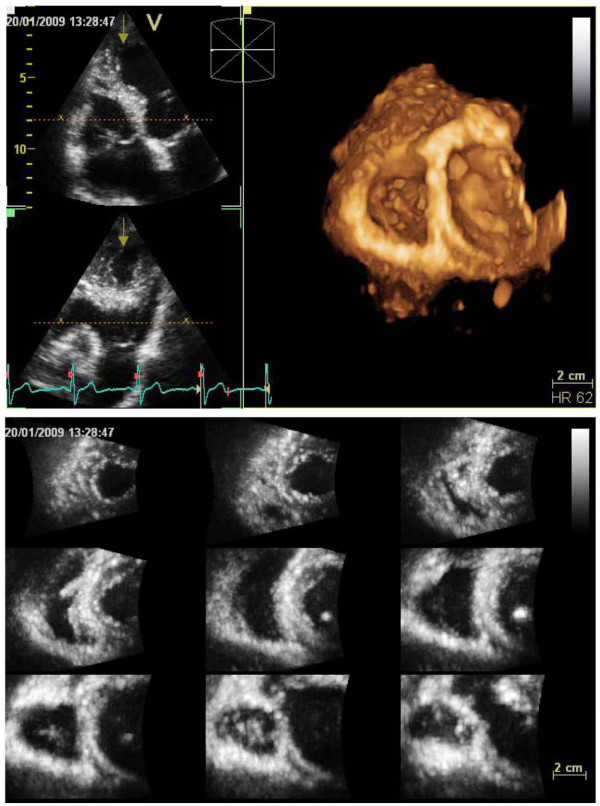
**Real-time 3-D echocardiography for quantitation of the right ventricle**. 2-D RV multiplane display and 3-D RV transversal plane during acquisition (upper panel) as well as 9-slice display after acquisition were used for quality check (lower panel).

A full-volume RV data-set was acquired from apical approach using harmonic imaging, with adjustment of image contrast, frequency, depth and sector size for adequate frame-rate and optimal RV border visualization [10]. Tricuspid valve, but not the entire right atrium, was included in the data set throughout the cardiac cycle. Gain was set higher than for usual 2-D images [10]. Respiratory maneuvers were applied for optimizing endocardial border visualization, especially when RV anterior wall could not be otherwise encompassed in the data set. Then, four ECG-gated subvolumes were acquired from consecutive cardiac cycles during breathholding to generate the full-volume data set. Similar to the left ventricle, peripheral labs were requested to perform quality check by 2-D RV multiplane display and 3-D RV transversal plane during acquisition as well as by 9-slice display immediately after acquisition (Figure 6).

Adequate 3-D data sets of left and right ventricles were stored digitally in raw-data format.

#### 2. Reading procedures (in the Echo Study Group)

For each reproducibility testing two independent observers analyzed off-line data sets (number range of video clips and images for each cardiac ultrasound technique ≥ 50) stored in the EchoPac BT 09. Table 3 lists the main parameters of each echo-Doppler modality selected for reproducibility analyses. According to recent EAE recommendations [2], these parameters were chosen on the basis of recognized characteristics including accuracy (i.e., validation against autopsy and/or reference techniques as cardiac magnetic resonance imaging), reproducibility, reliability and prognostic value in the clinical setting.

**Table 3:** Main echo-Doppler parameters selected for reproducibility analyses.

Echo-Doppler Modality/Technology Parameter	
**M-mode echo**	LV mass
	LV mass index (for BSA and height)
	Relative diastolic wall thickness *
	TAPSE

**2-D echo**	LV end-diastolic volume
	LV end-systolic volume
	LV EF
	LA volume
	LA volume index (for BSA)
	Aortic diameter at multiple levels
	RV diameters

**Doppler-derived diastolic function**	Transmitral E/A ratio
	E velocity deceleration time
	e' velocity of mitral annulus (septal and lateral)
	E/e' ratio

**Speckle Tracking Echocardiography**	Global longitudinal strain
	Global circumferential strain
	Global radial strain
	LV twisting

**3-D echo**	LV end-diastolic volume
	LV end-systolic volume
	LV EF
	RV end-diastolic volume
	RV end-systolic volume
	RV EF

BSA = body surface area, E = Transmitral E velocity e' = early diastolic velocity of the mitral, annulus, EF = Ejection fraction, LA = Left atrial, LV = Left ventricular, RV = Right ventricular, TAPSE = Tricuspid annular plane systolic excursion

°Relative wall thickness calculated as (SWT - PWT)/LVIDD where LVIDD = Left ventricular internal diameter at end-diastole, PWT = Posterior wall thickness, SWT = Septal wall thickness

All M-mode, 2-D echo and Doppler measurements were averaged over 3 consecutive cardiac cycles [2].

##### LV structure and function [2,5]

In order to obtain LV diameters, wall thickness and LV mass, measurements were taken using M-mode or direct 2-D echo measurements in parasternal long-axis view. LV (septal and posterior) wall thicknesses and internal diameters were measured approximately at the mitral valve leaflet tips perpendicularly to the long axis. LV end-diastolic (EDV) and end-systolic (ESV) volumes were measured in apical 4-chamber and 2-chamber views by modified Simpson' method to assess EF and averaged.

##### LA structure [2,5]

Two-dimensional estimation of LA volume was performed at LV end-systole in modified apical 4-chamber and 2-chamber views, by using both area-length and disc summation methods. When performing LA planimetry for LA volume calculation, the confluences of the pulmonary veins and LA appendage were excluded, and the mitral plane was drawn as a straight line connecting lateral and septal sides of the mitral annulus.

##### Aortic root and ascending aorta [2,6]

The aortic diameters were measured on 2-D echo images from a modified parasternal long-axis view at end-diastole (identified at QRS complex onset at ECG) according to both leading-edge to leading-edge and inner-edge to inner-edge methods perpendicularly to the long-axis of the aorta. Measurements were performed at the following multiple levels: sinuses of Valsalva, sino-tubular junction (transition between the sinuses of Valsalva and the tubular portion of the vessel), tubular ascending aorta (2-3 cm after the sino-tubular junction).

##### Doppler derived LV diastolic function [2,7]

To enhance reproducibility, the outer margins of the Doppler waveforms was taken into account for measurements. Caution was exerted when measuring E velocity deceleration time in presence of sinus tachycardia (overlapping of E and A waveforms), by prolonging the slope of E velocity into the A until the baseline. Pulsed TVI signal was measured at both septal and lateral sites of the mitral annulus and e' velocities were averaged to calculate E/e' ratio.

##### RV structure and function [2,5]

RV mid-cavity and basal RV diameter as well as RV longitudinal diameter were measured in 2-D echo apical 4-chamber view. TAPSE was measured on M-mode tracings of lateral tricuspid annulus excursion as recommended [5].

##### Speckle Tracking Echocardiography [8]

Commercially available acoustic-tracking software was applied on 2-D gray-scale images by tracking movements of "speckles" in the myocardial tissue, frame by frame throughout the cardiac cycle. The software is interactive (endocardial-cavity interface traced manually and epicardial tracing generated automatically) and rejects poorly tracked segments, allowing the observer to manual override its decision by visual assessment. The time of aortic valve closure (AVC) is marked (either automatically or manually) looking at the motion of the aortic valve laeflets in the apical long-axis view, which has therefore to be analyzed first and used as reference timing in all the other views. Each LV view is automatically divided into 6 myocardial segments. For the present assessment, peak negative longitudinal strain was measured from 6 segments in each of the 3 apical views (long-axis, 4- and 2-chamber) and global longitudinal strain (GLS) calculated as the average of individual peak strain before AVC. Global circumferential strain (GCS) and global radial strain (GRS) were obtained as the average of the regional values measured in the 6 myocardial segments of basal, middle and apical parasternal short-axis views. Basal to apical twisting was calculated as the net difference in LV rotation angle at the apical (counterclockwise, positive angles) and basal (clockwise, negative angles) short-axis plane occurring before AVC.

Longitudinal strain was quantified also by Automated Function Imaging (AFI), a software which applies STE principles "on-line" allowing longitudinal strain measurements during 2-D examination [11]. By using AFI, the endocardial-cavity interface is traced semi-automatically by marking only 3 points, 2 at the basal walls and 1 at the apex, in each apical view. Similar to STE measurements, peak negative longitudinal strain was calculated from 6 segments in each apical view and GLS calculated as the average of all the values. Reproducibility of longitudinal strain derived from AFI and STE were compared.

## RT3DE

### LV function

The software (4D AutoLVQ, GE Healthcare, Horten, Norway) has been validated against cardiac MRI and proved to have excellent agreement with other dedicated softwares for 3-D LV quantitation [12]. It provides automatic slicing of LV full-volume data-set, manual alignment of LV central longitudinal axis, LV reference point identification, automated identification of endocardial borders at both end-diastole and end-systole and final data set display. In order to increase accuracy, image gain settings can be preliminary adjusted to improve endocardial delineation. In the present assessment readers were required to apply semi-automatic detection of LV endocardial surface in order to obtain a dynamic surface-rendered LV cast from which EDV, ESV, EF, stroke volume and cardiac output were determined. Care was taken to verify that papillary muscles and endocardial trabeculae were included in LV cavity, and endocardial contour placed slightly outside the visible black-white interface [12]. In case of unsatisfactory verification, readers manually adjusted LV borders by placing additional points, with further refinement of boundary detection and a new data set display. The reproducibility of semi-automatic and manually adjusted LV measurements were compared.

### RV Function

The software (4D-RV function, version 2.6, TomTec Imaging Systems, Gmbh, Unterschleissheim, Germany) is clinically validated against cardiac MRI [10,13]. Every RV full-volume 3-D data set is automatically cropped in 3 standard planes (views): 4-chamber, coronal and sagittal. After optimizing each view according to anatomical landmarks (RV inflow and outflow), 3-D data sets can be manipulated by the reader with a series of translational, rotational and pivoting manoeuvres, for the reference line to pass through the center of tricuspid valve and RV apex in each view. End-diastolic (largest RV area) and end-systolic (smallest area) frames are then manually set in 4-chamber view. Point identification for mitral and tricuspid valve and LV apex are required. In the present assessment readers were required to trace endocardial border at end-diastole and end-systole for the 3 selected RV planes. Care was taken to trace endocardial border just outside the blood-tissue interface and papillary muscles, moderator band and endocardial trabeculae were included in RV cavity. These manually traced contours served for initiation of automated border detection algorithm. Frame-by-frame orrection of endocardial border was applied when needed. Measurements of RV EDV, ESV, EF and stroke volume and cardiac output were finally obtained.

#### 3. Statistics Plan

All statistical analyses will be performed by the Study Centre of the Italian Society of Cardiology.

Values will be reported as mean and standard deviation (SD). The reproducibility will be expressed as the coefficients of reproducibility (CR) and the mean percent errors (mean errors). CR for inter-observer and intra-observer measurements will be evaluated by Bland and Altman test [14]. The CR represents the limits of agreement within which 95% of the differences expected to be. Mean error will be derived as the absolute difference between the two sets of readings, divided by the mean of the readings. For intra-observer, the CR will be defined as the standard deviation (SD) of the difference from the mean of the repeated measurements divided by the mean response. The CR of inter-observer reproducibility will be defined as the SD of difference between the pairs of measurements obtained by the two readers, divided by the average of the means of each pairs of readings. For comparison of the mean errors of measurements, one-way analysis of variance (ANOVA) will be used.

The proportion of echo-Doppler measurements classified as abnormal (according to standardized cut.-off point values) using data from the two different readers will be reported, and the 2-by-2 cross-tabulation of findings from the two readings will be used to calculate the rate of intra-participant between-study reclassification rate. The Cohen's K statistic will be performed as measure of between-study agreement of index abnormality identified. Higher values of K indicate low rate of intra-participant between-study reclassification and, therefore, elevated between-study agreement. A two-sided P < 0.05 will be considered a marker of statistical significance. All statistical analyses will be performed using SAS 9.3.

## Discussion

### Implications

The improvement of imaging acquisition and measurements reproducibility of echo-Doppler is a mainstay to increase its clinical usefulness and reduce the need of repeated examinations in everyday clinical practice. This fundamental rule should be carefully taken into account also in each non-referral echo-lab, which should assess and re-test periodically its own internal reproducibility. Recommendations have established standardized procedures of performance, storage and reporting of echocardiographic studies [4] as well as echo laboratory standards and accreditation processes [15,16]. As recommended [1-3], quality control is of primary importance to reduce variability among institutions and operators. This issue becomes imperative when applying echo-Doppler in clinical trials. The establishment of an ECL plays a crucial role to reduce intra- and inter-observer variability of different echo-labs involved in a multicenter study. An ECL cannot eliminate the different sources of variability but it can ensure that the acquisition as well as the errors of measurements are controlled and do not occur randomly [17].

This report proposes a methodological approach which is consistent with related EAE recommendations [2,4] but possibly provides further refinements. In particular, specific requirements were addressed:

1. to the operators of peripheral labs during the acquisition processes of

a. 2-D echo (by performing dedicated views of left atrium and aorta),

b. STE (obtaining the best frame rate to track LV walls throughout the narrowest scan at the lowest possible depth),

c. quality check of RT3DE-derived LV and RV full-volume data set.

2. to the readers of an ECL for:

a. 2-D measurements (comparison of reproducibility between area-length and disc summation methods for LA volume, comparison between leading-edge and inner-edge methods for aortic size),

b. GLS (comparison between STE and AFI softwares),

c. RT3DE (comparison between automatic and manually adjusted LV measurements).

The results of these comparisons may contribute to optimize the approach to each measurement both for clinical purposes and for a given trial.

## Limitations

Limitations of the present project include the absence of testing biological (*day-to-day *or *test-retest*) and inter-vendor (*machine-to-machine*) reproducibility. This latter is critical for newer echo techniques and in particular for STE which has demonstrated relevant discrepancies by comparing different commercially available cardiac ultrasound systems [18]. While it remains an open question for either STE or RT3DE assessment of LV function, in our approach the use of a inter-changeable RT3DE software for the right ventricle is valuable, it being compatible with any kind of echo manufacture producing 3-D data sets.

## Perspectives

By this document the Study Group of Echocardiography of the Italian Society of Cardiology proposes itself as an ECL with its standardized procedures of echo-Doppler acquisition and reading. Any quality control process, such as the one described for ECL readings, has a two-fold value for cardiologist involved. In fact, the participating echo laboratories are not only involved in important accuracy-related issues, but can also benefit in terms of training and competence improvement. This may be particularly relevant as a teaching tool for fellows in training. These procedures can be suggested also for utilization in non-referral echocardiographic laboratories as a self check of quality, to improve reproducibility and increase clinical consistency of echo-Doppler assessment. The analyses of each different (standard and advanced) echo-Doppler modalities/technologies are planned to be presented/submitted for subsequent documents which will show the results of intra- and inter-observer variability.

## Competing interests

The authors declare that they have no competing interests.

## Authors' contributions

MG and SN conceived of the study and participated in its design and coordination, drafted the manuscript and performed echo-Doppler readings, SM, MAL, PI and DM participated in the study design and coordination and performed echo-Doppler readings, DM, ADA, PB, AS, RE, GM and AS participated in the study coordination, performed echo scans and readings and revised the manuscript, EA, LPB, RM (responsible also of statistical analysis), PPF, GM and PNM participated in the study design and revised the manuscript. All the authors read and approved the final manuscript.
